# Prevention and treatment of natural products from Traditional Chinese Medicine in depression: Potential targets and mechanisms of action

**DOI:** 10.3389/fnagi.2022.950143

**Published:** 2022-07-18

**Authors:** Ming-Chao He, Rui Feng, Jing Wang, Shi-Hui Xia, Yong-Jun Wang, Yan Zhang

**Affiliations:** ^1^Spine Disease Research Institute, Longhua Hospital, Shanghai University of Traditional Chinese Medicine, Shanghai, China; ^2^Shanghai Geriatric Institute of Chinese Medicine, Shanghai, China

**Keywords:** Traditional Chinese Medicine, depression, transcription factor, signaling pathway, action target

## Abstract

The molecular pathology involved in the development of depression is complex. Many signaling pathways and transcription factors have been demonstrated to display crucial roles in the process of depression occurrence and development. The multi-components and multi-targets of Traditional Chinese Medicine (TCM) are uniquely advantageous in the prevention and treatment of chronic diseases. This review summarizes the pharmacological regulations of natural products from TCM in the prevention and treatment of depression from the aspects of transcription factors (CREB, NF-κB, Nrf2) and molecular signaling pathways (BDNF-TrkB, MAPK, GSK-3β, TLR-4).

## Introduction

Depression is a common mental disease and its main clinical features include not only mental symptoms such as persistent depression, loss of interest, guilt, and low self-esteem, but also physical symptoms such as lack of energy, fatigue, insomnia, and difficulty in concentrating. In the most severe cases, depression can lead to suicidal tendencies. Globally, about 5% adults suffer from depression, which rises to the most common disabling condition by 2030 (Barnett, 2019; Herrman et al., 2022). The causes of depression are complex such as interaction of social, psychological, and physical factors. Besides, depression is often associated with other mental and chronic illnesses (Wolock et al., 2020). Several theories based on biological studies have been developed to clarify the pathogenesis of depression, including the monoamine hypothesis (Shao and Zhu, 2020), the hypothalamic-pituitary-adrenal axis (HPA) dysfunction theory (Eliwa et al., 2021), the neuroplasticity hypothesis (Boku et al., 2018), the inflammation theory (Gałecki and Talarowska, 2018), and the neurodegeneration hypothesis (Tauil et al., 2021).

Monoamine neurotransmitters are a group of neurotransmitters secreted mainly in the brain and the adrenal glands, including dopamine, norepinephrine, epinephrine, and 5-hydroxytryptamine. Since the antihypertensive drug reserpine induced depression and isoniazid improved depressed mood, it was demonstrated that the reduced level of 5-hydroxytryptamine in the central nervous system could contribute to the development of depression (Coppen et al., 1965). Subsequently, the monoamine hypothesis was proposed which is the prominent theoretical basis for most antidepressant drugs.

Currently, pharmacological therapy is still the mainstay of clinical practice for patients with depression. The majority of antidepressants now in use, such as selective serotonin reuptake inhibitors (SSRIs), monoamine oxidases inhibitors (MAOIs), and tricyclic antidepressants (TCAs), rely on modulating monoamine neurotransmission as the primary pathway (Harmer et al., 2017; Wang et al., 2019). However, clinical antidepressants generally have a series of drawbacks, such as the slow onset of action, serious adverse reactions, high price, and the tendency to relapse after discontinuation of the drug. In other words, they still could not meet clinical needs for both efficacy and side effects. In contrast, natural products consist of many bioactive components that can act on multiple targets and effectively avoid the side effects produced by single link action. Its unique efficacy also facilitates the exploration of the pathogenesis of depression from various theoretical perspectives, which makes it possible to treat depression as well as complications associated with depression more effective. As a result, researchers are most interested in exploring antidepressant ingredients from natural products, and some groups have made substantial progress in depression treatment. This review summarized the effects of active naturally occurring constituents on the important targets and the relevant signaling pathways for the prevention and treatment of depression, as well as provided a basis for the research and development of novel antidepressants characterized with rapid action.

## Transcription factors

Transcription factors such as cyclic-adenosine monophosphate (cAMP) response element-binding protein (CREB), nuclear factor kappa B (NF-κB), and nuclear factor erythroid 2-related factor 2 (Nrf2) play an important role in the development of depression, and they could also interact with other transcription factors. Therefore, the regulation on the activation or inhibition of transcription factors might be an effective strategy to combat depression, and actually, emerging pieces of evidence have demonstrated that natural products could exert anti-depressive effects by regulating transcription factors.

### Cyclic-adenosine monophosphate response element-binding protein

cAMP response element-binding protein is a member of the basic leucine zipper (bZIP) family of transcription factors and has the ability to stimulate PKA *via* the cAMP response element, thereby regulating the transcription of target genes (Zhou et al., 2021). CREB is expressed in many tissues and plays a very important regulatory role in the nervous system. CREB not only promotes the proliferation of neuronal precursors and the mature differentiation of developing neurons, but also plays a key role in multiple signaling pathways related to neuroplasticity, learning, and memory (Wu et al., 2020). Many drugs can improve depressive behaviors by upregulating the expression of CREB in different regions of the brain. Thus, targeting and regulating CREB is proposed to have important implications for the treatment of depression and the rational development of new drugs (Carlezon, et al., 2005).

Crocin is one of the main active ingredients in gardenia. Clinical studies have shown that the therapeutic effect of crocin on mild to moderate depression is comparable to that of imipramine (Zhang et al., 2018). An animal study consistently reported that crocin played an antidepressant role in mice with chronic unpredictable mild stress (CUMS) and corticosterone-induced cell models by enhancing synaptic plasticity and improving neuronal survival (Lu et al., 2020). The underlying mechanism was partially attributed to the fact that crocin significantly increased CREB phosphorylation. In addition, a gardenia yellow pigment is a group of compounds with a shared structure of crocin, which activated ERK, CREB, and the downstream effector, thereby exerting a rapid antidepressant effect in the learned helplessness paradigm (Wu et al., 2016).

### Nuclear factor-kappa B

Nuclear factor-kappa B (NF-κB), found in almost all animal cells, is a key transcription factor. NF-κB binds to the inhibitor of nuclear factor kappa-B kinase (I-κB) in unstimulated cells and is isolated in the cytoplasm. Activation of I-κB kinase (IKK) under various stimuli leads to the phosphorylation of I-κB, which leads to its degradation by the proteasome and releases NF-κB into the nucleus. Subsequently, NF-κB binds to target sequences in the nucleus and activates gene transcription (Koppe et al., 2019). NF-κB regulates the expression of several genes and is a major regulator of immunity and inflammation in injury and infection states. The over-activation of NF-κB is strongly associated with many diseases such as rheumatoid arthritis, cancer, infectious shock, and inflammatory responses in the brain. It has been found in recent studies that patients with chronic inflammation who suffer from depression have higher levels of circulating pro-inflammatory factors compared to those with common chronic inflammation (Ménard et al., 2017). This suggests that inflammatory factors such as tumor necrosis factor α (TNF-α), interleukin-1β (IL-1β), and interleukin-6 (IL-6) may be involved in the development of mood changes that are characteristic of clinical depression (García-García et al., 2022). Recently, a study performed on depressive mice found that the inflammatory responses in the hippocampus and cortex were closely linked to NF-κB (Fronza et al., 2022), suggesting that the NF-κB signaling pathway might play a key role in the depressive process. Therefore, inhibition of the NF-κB signaling pathway may be one of the strategies to treat inflammation-induced depression.

Icariin (ICA) is the main active component of flavonoids in the Herba Epimedii that has a wide range of pharmacological effects, including anti-inflammatory (Luo et al., 2022), antioxidant (Li et al., 2022), antidepressant (Xu et al., 2020), etc. In addition, ICA was found to improve fur status and increase body weight, sucrose preference, and activity time in the forced swimming test in depressed rats induced by CUMS. Mechanistic studies confirmed that ICA alleviated oxidative stress injury and inhibited neuroinflammation in the hippocampus of depressed rats by inhibiting NF-κB protein expression and the activation of the NLR family pyrin domain containing 3 (NLRP3) inflammasome (Liu et al., 2015). Similarly, the combination of geniposide and eleutheroside B improved lipopolysaccharides (LPS)-induced depressive-like behavior in mice and downregulated the protein expression level of NF-κB in hippocampal tissues. Moreover, the combination of geniposide and eleutheroside B suppressed neuroinflammation by inhibiting NF-κB activation and decreasing the expression of inflammatory factors (Zhang et al., 2021).

### Nuclear factor erythroid 2-related factor 2

Nuclear factor erythroid 2-related factor 2 belongs to the bZIP family of transcription factors and is a major regulator of oxidative stress. It maintains a dynamic redox balance by transcribing various antioxidant enzymes to protect the organism from damage (Sivinski et al., 2021). Nrf2 is important in the regulation of excessive inflammatory responses caused by oxidative stress in the brain. Nrf2 reversed depressive symptoms through an anti-inflammatory mechanism and chronic inflammation caused by Nrf2 deficiency led to a depressive-like phenotype, making Nrf2 a possible target for the development of novel antidepressants (Muhammad et al., 2022).

It has been shown that sulforaphane, a potent agonist of Nrf2, ameliorated depression-like behavior in LPS-induced mice, decreased serum levels of TNF-α and IL-10, and inhibited activation of microglia. Sulforaphane may protect neurons from oxidative stress through the Keap1-Nrf2 pathway and reduce neuroinflammation, thereby exerting antidepressant effects (Zhang et al., 2017). In addition, tanshinone IIA and ginsenoside-Rg1 also suppressed microglial activation, reduced excessive release of pro-inflammatory cytokines, and exerted neuroprotective effects by increasing the transcriptional activity of Nrf2 (Cai et al., 2017; Fan et al., 2018).

## Signal pathways

Brain-derived neurotrophic factor (BDNF)-tropomyosin receptor kinase B (TrkB) pathway, mitogen-activated protein kinase (MAPK) pathway, glycogen synthase kinase-3 (GSK-3) pathway, toll-like receptor 4 (TLR4) pathway, etc. are involved in the pathological process of depression. Therefore, it is highly feasible to intervene by targeting these important signaling pathways in the pathogenesis of depression. The targeting of signaling pathways by natural products from Traditional Chinese Medicine (TCM) provides a theoretical basis for the research and development of antidepressants.

### Brain-derived neurotrophic factor-tropomyosin receptor kinase B pathway

Brain-derived neurotrophic factor is a secreted protein member of the neurotrophic factor family with effects on the central nervous system. It exerts a key role in neuronal survival, differentiation, and plasticity. BDNF is processed in neurons, transported to the synaptic terminal before being released, and then bound to TrkB as a dimer form (Caviedes et al., 2017). BDNF and its mediated signaling pathways may exert antidepressant effects through altering synaptic plasticity as well as affecting neural circuit formation and neuronal survival.

Naringenin (4′,5,7-trihydroxyflavanone) is one of the main flavonoids found in the peels of citrus fruit (Dong et al., 2022). Naringenin was found to promote the expression of BDNF in the hippocampus of CUMS mice. K252a, an inhibitor of TrkB, eliminated the antidepressant-like effect of naringenin in the sucrose preference test and novelty-suppressed feeding test. This suggests that activation of the BDNF signaling pathway in the hippocampus may mediate, at least in part, the antidepressant-like effect of naringenin. In addition, long-term administration of naringenin reversed CUMS-induced prolongation of first feeding latency, suggesting that naringenin may also improve anxiety-related behaviors (Yi et al., 2014). 20(S)-protopanaxadiol (PPD), a well-known medicinal herb widely used in East Asia, is a basic aglycone of the tetracyclic triterpenoid saponins found in Panax ginseng (Peng et al., 2019). PPD significantly improved chronic social defeat stress (CSDS)-induced depressive-like behavior. It may exert antidepressant-like effects by modulating neurotransmitter and corticosterone levels, improving oxidative stress, enhancing the PI3K/Akt/mTOR-mediated BDNF/TrkB pathway, and promoting the normalization of HPA function (Jiang et al., 2019). Analogously, it was found that piperine inhibits monoamine oxidase activity, elevates 5-HT and BDNF levels in brain tissue, and regulates the HPA axis (Mao et al., 2011). This suggests that the antidepressant-like effects of piperine may be mediated through inhibition of oxidative stress and upregulation of BDNF expression (Mao et al., 2014).

### Mitogen-activated protein kinase pathway

The MAPK signaling pathway controls complex cellular events such as proliferation, differentiation, and apoptosis. A growing number of studies have shown that ERK1/2 and p38MAPK play a key role in a variety of psychiatric disorders such as depression. Various chronic stresses have been reported to cause a decrease in p-ERK and an increase in p-p38MAPK in the prefrontal cortex and hippocampus of experimental rodents (Li et al., 2017; Han et al., 2019). Furthermore, the acute administration of a MAPK pathway inhibitor increases depressive-like behavior in mice and blocks the effects of antidepressants. Therefore, modulation of the MAPK signaling pathway may be one of the targets to combat depression.

Triptolide reversed the depressive-like behavior produced by depression comorbidity in the chronic pain rat model induced by spinal nerve ligation. This may be related to its inhibition of elevated levels of p38 phosphorylation in the hippocampus and activation of microglia, which significantly attenuated neuroinflammation caused by nerve injury in the rat hippocampus (Hu et al., 2017). Similarly, baicalein had an antidepressant-like effect in experimental animal models. It has been shown that baicalein was able to significantly increase p-ERK1/2 levels in the hippocampus of CUMS rats. Thus, this antidepressant-like effect is at least partially related to ERK-mediated neurotrophic action (Xiong et al., 2011).

### Glycogen synthase kinase-3β pathway

Glycogen synthase kinase-3 is a highly conserved family of protein kinase that acts by phosphorylating amino acid residues of serine/threonine (Panagi et al., 2020). GSK-3 is divided into two subtypes, GSK-3α and GSK-3β (Chandra et al., 2021). Phosphorylation at ser21 of GSK-3α or ser9 of GSK-3β can significantly inhibit its activity. It is known that the phosphatidylinositol 3-kinase (PI3K)–Akt pathway plays an important role in the regulation of GSK-3β activity, and Akt may directly phosphorylate GSK-3β and inactivate it. It has been proposed through animal models of depression that inhibition of GSK-3β may contribute to the antidepressant effect (Ebeid et al., 2021). Another clinical study noted that the kinase activity of GSK-3β was significantly in the ventral prefrontal cortex of patients with major depression, while the activity of Akt was reversed (Karege et al., 2007). As such, the GSK-3β signaling pathway may be an applicable therapeutic target and pathway for depression treatment.

Isorhynchophylline (IRN), isolated from Uncaria rhynchophylla, is its main active component. In the course of CUMS, chronic administration of IRN attenuated depressive-like behaviors in mice. Also, IRN increased the ratio of p-GSK-3β (Ser9)/GSK-3β and p-Akt (Ser473)/Akt in the hippocampus and cerebral cortex of mice. This indicated that the effect of IRN on improving CUMS-induced neuroinflammatory and depression-like behaviors was directly related to the regulation of the GSK-3β pathway (Yuan et al., 2009). Dihydromyricetin (DHM) is a flavonoid natural product that has a variety of pharmacological effects, including anti-inflammatory, antioxidant, and antitumor effects. DHM significantly improved CUMS-induced and LPS-induced depression-like behavior in mice and produced faster antidepressant-like effects than the typical antidepressant venlafaxine. DHM upregulated the phosphorylation levels of ERK1/2 and GSK-3β, in both hippocampal tissues and cultured hippocampal cells. The results suggested that this effect may be achieved through activation of the ERK1/2-CREB pathway, as well as inhibition of GSK-3β and neuroinflammation (Ren et al., 2018).

### Toll-like receptor-4 pathway

Toll-like receptor (TLR) signaling plays an essential role in the innate immune system response. The TLR family includes at least 13 members, and TLR-4 is one of the important pattern recognition receptors. It recognizes a variety of molecular patterns associated with microbial pathogens, such as LPS and heat shock proteins (HSPs), and modulates both innate and acquired immune responses (Paudel et al., 2020). TLR4 is activated upon stress or damage and causes an inflammatory response with production of inflammatory cytokines such as TNF-α, IL-1β, IL-6, etc. (Tan et al., 2017). Recent studies have found that protein expression and mRNA expression of TNF-α, IL-1β, and IL-6 are significantly elevated in the brains of depressed patients who have committed suicide (Pandey, 2017). Besides, several studies suggested that IL-1β-mediated neuroinflammatory responses promote depressive-like behavior in mice (Cheng et al., 2018). Oligomerization and activation of the NLRP3 inflammasome lead to cysteinyl aspartate specific proteinase 1 (caspase-1) activation, which in turn cleaves the pro-IL-1β to mature IL-1β and releases it (Latz et al., 2013; Kaufmann et al., 2017). Therefore, modulation of the TLR4 signaling pathway may be one of the most effective strategies to combat depression.

While ameliorating the depression-like behavior of depressed model rats, it was found by hematoxylin-eosin staining that salvianolic acid B attenuated the nuclear condensation and acidophilic degeneration of neurons in the hippocampus caused by LPS. In accordance, salvianolic acid B inhibited microglial activation and decreased mRNA expression of IL-1β and IL-6. In the meantime, salvianolic acid B inhibited NLRP3 inflammasome activation and significantly decreased protein levels of the NLRP3 and caspase-1. The results suggested that salvianolic acid B may produce neuroprotective and antidepressant effects by inducing the clearance of excessive NLRP3 (Jiang et al., 2017). Similarly, pre-treatment with muscone reduced tissue content and protein expression of IL-1β in the prefrontal cortex of mice. Assessment of IL-1β upstream signaling pathways revealed that muscone inhibited the activation of NLRP3 inflammasome through downregulation in protein expression of caspase-1 and NLRP3 and suppressed the expression of key proteins in the TLR4/MyD88 pathway of the prefrontal cortex of mice with LPS-induced depression. It is suggested that muscone may alleviate IL-1β-related CNS inflammation through TLR4/MyD88 and TLR4/NLRP3 pathways, thereby attenuating LPS-induced depression-like behaviors (He et al., 2020). In addition, both andrographolide (Geng et al., 2019) and allicin (Gao et al., 2019) could ameliorate excessive inflammation status by inhibiting the activation of NLRP3 and corresponding components, thereby exerting antidepressant effects.

## Discussion

**FIGURE 1:**
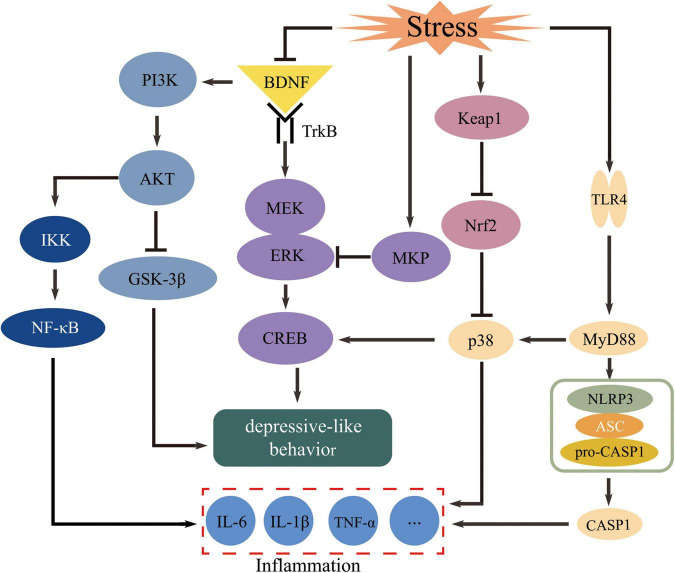
Transcription factors and signaling pathways potentially regulated by natural products from Traditional Chinese Medicine (TCM) in the prevention and treatment of depression.

The pathogenesis of depression is complex, with involvement of multiple transcription factors and molecular signaling pathways (Figure 1). Almost all clinical antidepressants, such as SSRIs, MAOIs, and TCAs, were discovered assuming the salience of the monoamine hypothesis. However, the overall clinical cure rate is less than 50% (Cipriani et al., 2016), moreover, there are many unwanted adverse reactions to the drugs used in clinical practice. Thus, the development of more effective and safer antidepressants has become a major concern today. Meanwhile, a diversity of multi-target strategies is proposed as successful antidepressants for the future. It has a long history with traditional experiences for TCM in the treatment of depression, and its effects are more sustained, moderate, and stable, making TCM more suitable for the long-term pharmacological management of depression. While, the lack of clear quality control standards to ensure the stability and consistency of the chemical composition especially the bioactive components in TCM will restrict its clinical application.

In the exploration of new antidepressants, natural products have always played an essential role due to their multi-components and multi-pharmacological effects. Natural products from TCM could improve the organism’s ability to resist disease while benefiting the overall internal environment. Focus on the targeting of medications while concentrating on overall regulation might make a better treatment for depression. The activation or inhibition of transcription factors including CREB, NF-κB, and Nrf2 exert crucial regulation involved in the antidepressant process of TCM. Considering the impaired neuroplasticity also appears as a key feature of depression, TCM that could restore neuronal plasticity and reduce neural damage induced by stress as well as other negative stimuli is more likely to preserve against depression by regulating cAMP signaling pathway. In addition, TCM targeting BDNF-TrkB pathway, MAPK pathway, GSK-3β pathway, and TLR-4 pathway, have shown to be effective in alleviating depressant symptoms. In a word, natural products with less side effects and kinds of action mechanisms might be a vital choice to be clarified for developing novel medication therapies (Table 1). At present, most of the studies on the targeted treatment of depression by natural products are still in the stage of preclinical experiments, and relevant clinical studies are lacking. Besides, the exact relationship between the active ingredients of natural products and the pathogenesis of depression is poorly studied. In-depth research on the causes and mechanisms of depression, elucidating the exact active ingredients of natural products and their molecular pharmacological effects in the prevention and treatment of depression, as well as the development of relevant target drugs, will be the key points and difficulties for future research.

**TABLE 1:** Transcription factors and signaling pathways involved in prevention and treatment of natural products from Traditional Chinese Medicine (TCM) on depression.

Targets	Natural products from TCM
CREB	crocin
	gardenia yellow pigment
NF-κB	icariin
	combination of geniposide and eleutheroside B
Nrf2	sulforaphane
	tanshinone IIA
	ginsenoside-Rg1
BDNF-TrkB pathway	naringenin
	20(S)-protopanaxadiol
	piperine
MAPK pathway	triptolide
	baicalein
GSK-3β pathway	isorhynchophylline
	dihydromyricetin
TLR-4 pathway	salvianolic acid B
	muscone
	andrographolide
	allicin

## Author contributions

M-CH and RF drafted the manuscript. S-HX, JW, and Y-JW prepared the figure and table. YZ conceived the topic and revised the manuscript. All authors contributed to the article and approved the submitted version.

## Conflict of interest

The authors declare that the research was conducted in the absence of any commercial or financial relationships that could be construed as a potential conflict of interest.

## Publisher’s Note

All claims expressed in this article are solely those of the authors and do not necessarily represent those of their affiliated organizations, or those of the publisher, the editors and the reviewers. Any product that may be evaluated in this article, or claim that may be made by its manufacturer, is not guaranteed or endorsed by the publisher.
